# Laboratory-scale characterization of saturated soil samples through ultrasonic techniques

**DOI:** 10.1038/s41598-020-59581-4

**Published:** 2020-02-21

**Authors:** Hongwei Liu, Pooneh Maghoul, Ahmed Shalaby

**Affiliations:** Department of Civil Engineering, University of Manitoba, 75A Chancellors Circle, Winnipeg, MB R3T 5V6 Canada

**Keywords:** Engineering, Civil engineering

## Abstract

The propagation of poroelastic waves in a soil specimen is dependent on the physical and mechanical properties of the soil. In the geotechnical practice, such properties are obtained through in-situ geotechnical testings or element soil testings in the laboratory. These methods require almost advanced equipment and both testing and sample preparation may be expensive and time-consuming. This paper aims to present an algorithm for a laboratory-scale ultrasonic non-destructive testing to determine the physical and mechanical properties of saturated soil samples based on the distribution of stress waves. The ultrasonic setup, in comparison to most conventional soil lab testing equipment, is low-cost and non-invasive such that it reduces the soil disturbance. For this purpose, a poro-elastodynamic forward solver and differential evolution global optimization algorithm were applied to characterize the porosity, density, and other mechanical properties for a soil column. The forward solver was developed based on a semi-analytical solution which does not require intensive computational efforts encountered in standard numerical techniques such as the finite element method. It was concluded that the proposed high-frequency ultrasonic technique characterizes effectively the saturated soil samples based on the output stress wave measured by the receiver. This development makes geotechnical investigations time-efficient and cost-effective, and as such more suited to applications in remote areas.

## Introduction

Characterizing foundation soils is the first step in design and construction of civil infrastructure. The measurement of physical and mechanical properties of soils (e.g shear wave velocity, compression wave velocity, density and porosity) requires intensive in-situ or laboratory tests, which can be time consuming and costly. Soil samples, especially from projects in remote areas, are required to be transported to a geotechnical laboratory for various tests. This can cause the disturbance of soil samples, and laboratory tests on disturbed samples may lead to erroneous conclusions.

The laboratory methods for measuring the shear wave velocity of soil samples include the resonant column test, bender element test among others. However, there is no established standard developed for the interpretation of the dynamic test results^1^. The bender element method was developed in the 1980s and its simplicity is widely recognized: one transducer is placed at one end of a soil specimen for the generation of stress waves; one receiver is placed at the other end to record the induced stress waves. Various interpretation methods have been proposed in the past. The shear wave velocity can be calculated from the time difference between the input and output waves by assuming the absence of reflected or refracted waves^2^. However, it is well known that the identification of the arrival time of the output wave is subjective^1^. Other signal processing techniques such as the cross-correlation of the input and output stress waves^3^ and the second arrival of the output wave^4^ are based on the peak values of the stress wave for the estimation of the shear wave velocity. Some other methods (e.g. *π*-point identification^5^ and frequency spectral analysis^6^) are used for estimating the relation between the phase angle and shear wave velocity in the frequency domain.

The elastodynamic theory has been also used by several researchers^7–9^ through the finite difference, finite element, and discrete element methods to interpret the output stress waves. The elastodynamic algorithm assumes that the domain is composed of solid materials. Under a dynamic load, the generated P waves and S waves penetrate into different layers of a soil and the reflected waves received at the receiver can be used to determine the soil strata. However, the estimation of the shear wave velocity is still based on the simple signal processing techniques. In addition, in elastodynamic algorithms, the effect of porous structure of soil layers and pore water in dynamic responses of geomaterials is neglected. In fact, the wave propagation in porous soil layers can be better represented by using dynamic poroelastic models instead of elastodynamic models, especially in fully saturated soils in which the pore water can significantly attenuate the stress waves, and in high frequency regimes. The dynamic poroelastic models consider the coupling effect between the pore water and solid skeleton, which induces three types of waves (fast P wave, slow P wave, and S wave in the solid skeleton). Under an impact load, those three waves travel at different speeds, which are captured by the receiver placed at the end of the soil specimen in an ultrasonic setup.

The problem of dynamic poroelasticity^10,11^ has been solved using various analytical and numerical methods. A direct boundary element approach for solving three-dimensional problems of dynamic poroelasticity in the time domain was developed by^12^. Such a technique was based on an integral equation formulation in terms of solid displacements and fluid stress. The 2D and 3D fundamental solutions of dynamic poroelasticity was further developed by^13–16^. The solutions were obtained in both time and Laplace transform domain, and can be recovered to elastodynamics and steady-state poroelasticity. In layered saturated media, similar approaches have been reported by^17,18^. Other than the boundary element method, the finite element method has also been applied by^19^. The finite difference method is also used to simulate the wave propagation in heterogeneous poroelastic media by^20^.

In a conventional geotechnical apparatus used to determine the dynamic properties of a soil specimen, the focus is mainly on the estimation of shear wave velocity and the interpretation method is mostly based on the time interval difference between the input and output stress waves. To the best of our knowledge, there is currently no laboratory-scale ultrasonic setup which is able to determine a range of physical and mechanical properties of a soil sample. Furthermore, the development of cheaper, faster and portable means of soil characterization may significantly lower the cost of overall soil testing, and make better assessments possible in sensitive locations.

This paper aims to present an ultrasonic-based poroelastodynamic algorithm, which can be used in an ultrasonic setup to determine a range of physical and mechanical properties of a soil sample such as shear wave velocity, compression wave velocity, density and porosity. Such a setup can also be used for in-situ geotechnical investigation on extracted soil samples. In this algorithm, the poro-elastodynamic forward solver for the characterization of soil samples in high frequency regimes is developed using the spectral element method. Such a meshless semi-analytical technique reduces significantly the computational efforts by avoiding unnecessary calculations for the entire domain. Instead, only the response at the receiver location is calculated, which will then be used during the optimization process. A robust global optimization algorithm is then applied to predict the soil properties given the stress signal measured by the receiver.

## Problem Statement

**Figure 1:**
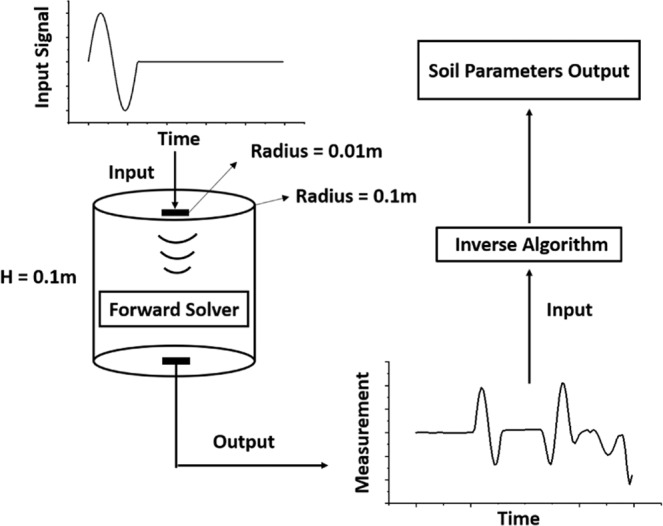
General schematic of the problem.

A general schematic of the problem is illustrated in Fig. 1. The domain is composed of a saturated porous medium. The transmitter located at one end of the sample generates the stress waves which travel through the specimen and is received by a receiver at the other end of the sample. The soil properties (Young’s modulus, Poisson’s ratio, density and porosity) will be captured by the proposed solver using the distribution of transmitted stress waves.

## Dynamic Poroelastic Forward Solver

By assuming the infinitesimal deformation of solid skeleton, the dynamic poroelastic governing equations are written as follows:

1a $$\mu {u}_{i,jj}+({\lambda }_{c}+\mu ){u}_{j,ji}+\alpha M{w}_{j,ji}=-\rho {b}_{i}+\rho \ddot{{u}_{i}}+{\rho }_{f}{\ddot{w}}_{i},$$

1b $$\alpha M{u}_{j,ji}+M{w}_{j,ji}=-f+{\rho }_{f}{\ddot{u}}_{i}+m{\ddot{w}}_{i}+b{\dot{w}}_{i},$$

where *u* is the displacement vector of the solid skeleton; *w* is the fluid displacement relative to the solid skeleton; *λ* and *μ* are the Lamé constants; *α* is the Biot coefficient; *p* is the pore-water pressure; *M* is 1/$(\frac{\phi }{{K}_{f}}+\frac{\alpha -\phi }{{K}_{s}})$ in which *K*_*f*_ is the bulk modulus of the fluid; *K*_*s*_ is the bulk modulus of the solid skeleton and *ϕ* is the porosity. *λ*_*c*_ = *λ* + *α*^2^*M*; *m* = *ρ*_*f*_*β*∕*ϕ* in which *β* is the tortuosity which is used to describe the diffusion properties in porous media, and *ρ*_*f*_ is the density of pore-water, taken as 1000 *kg*∕*m*^3^. The drag-force damping coefficient *b* is calculated as^21^:

2 $$b=\eta /\kappa \ F,$$

where *η* is the fluid dynamic viscosity and *κ* is the permeability coefficient; *F* is the viscous correction factor^22^:

3 $$F(\omega )=\sqrt{1+\frac{i}{2}{M}_{s}\frac{\omega }{{\omega }_{c}}},{\omega }_{c}=\frac{\eta \phi }{2\pi \beta {\rho }_{f}\kappa },$$

in which *M*_*s*_ is taken as 1; $i=\sqrt{-1}$ and *ω* is the angular frequency.

The governing equations can be written in frequency domain through the Fourier transform by performing convolution with *e*^−*iωt*^ in which $i=\sqrt{-1}$; *ω* is the frequency and *t* denotes time variable. The governing equations in Laplace domain can be obtained by replacing *ω* with  − *is* where *s* is the Laplace variable.

To obtain the analytical solution, the Helmholtz decomposition is used to decouple the P and S waves. The displacement vector is usually expressed in terms of a scalar potential (*ϕ*) and a vector potential ($\overrightarrow{\psi }=[{\psi }_{r},{\psi }_{\theta },{\psi }_{z}]$), as shown in Eqs. (4a and 4b). In axisymmetric conditions, only the components in *r* and *z* directions are considered. Since P wave exits in solid skeleton and fluid, two P wave potentials are used, *ϕ*_*s*_ and *ϕ*_*f*_, respectively.

4a $$\overrightarrow{u}(r,z)=\nabla {\phi }_{s}(r,z)+\nabla \times \overrightarrow{{\psi }_{s}}(r,z)\ and\ \nabla \cdot \overrightarrow{{\psi }_{s}}(r,z)=0,$$

4b $$\overrightarrow{w}(r,z)=\nabla {\phi }_{f}(r,z)+\nabla \times \overrightarrow{{\psi }_{f}}(r,z)\ and\ \nabla \cdot \overrightarrow{{\psi }_{f}}(r,z)=0.$$

The governing equations in frequency domain in terms of potentials are finally obtained as shown in Eqs. (5a, 5b, 5c and 5d):

5a $$({\lambda }_{c}+2\mu ){\nabla }^{2}{\widehat{\phi }}_{s}(r,z)+\alpha M{\nabla }^{2}{\widehat{\phi }}_{f}(r,z)=-\rho {\omega }^{2}{\widehat{\phi }}_{s}(r,z)-{\rho }_{f}{\omega }^{2}{\widehat{\phi }}_{f}(r,z),$$

5b $$-\mu {\nabla }^{2}{\widehat{\overrightarrow{\psi }}}_{s}(r,z)=\rho {\omega }^{2}{\widehat{\overrightarrow{\psi }}}_{s}(r,z)+{\rho }_{f}{\omega }^{2}{\widehat{\overrightarrow{\psi }}}_{f}(r,z),$$

5c $$\alpha M{\nabla }^{2}{\widehat{\phi }}_{f}(r,z)+M{\nabla }^{2}{\widehat{\phi }}_{f}(r,z)={-\omega }^{2}({\rho }_{f}{\widehat{\phi }}_{f}(r,z)+{\rho }_{m}{\widehat{\phi }}_{f}(r,z)),$$

5d $$0={\rho }_{f}{\omega }^{2}{\widehat{\overrightarrow{\psi }}}_{s}(r,z)+{\rho }_{m}{\omega }^{2}{\widehat{\overrightarrow{\psi }}}_{f}(r,z),$$

where *ρ*_*m*_ = *m* − *ib*∕*ω*; $\widehat{\ }$ represents the terms in frequency domain.

### Solution of dilation wave (P waves) using eigen decomposition

The equations in terms of P wave potentials (Eqs. (5a) and (5b)) in a matrix form is shown as:

6 $$\mathop{\underbrace{\left[\begin{array}{cc}{\lambda }_{c}+2\mu  & \alpha M\\ \alpha M & M\end{array}\right]}}\limits_{{K}_{P}}\,\left\{\begin{array}{c}{\nabla }^{2}{\widehat{\phi }}_{s}(r,z)\\ {\nabla }^{2}{\widehat{\phi }}_{f}(r,z)\end{array}\right\}={-\omega }^{2}\mathop{\underbrace{\left[\begin{array}{cc}\rho  & {\rho }_{f}\\ {\rho }_{f} & {\rho }_{m}\end{array}\right]}}\limits_{{\rm{M}}}\,\left\{\begin{array}{c}{\widehat{\phi }}_{s}(r,z)\\ {\widehat{\phi }}_{f}(r,z)\end{array}\right\}.$$

It can be seen from Eq. (6) that ${\widehat{\phi }}_{s}$ and ${\widehat{\phi }}_{f}$ are coupled in the governing equations. The diagonalization of such a matrix is required to decouple the system. The Eq. (6) is then rearranged into:

7 $$\left\{\begin{array}{c}{\nabla }^{2}{\widehat{\phi }}_{s}(r,z)\\ {\nabla }^{2}{\widehat{\phi }}_{f}(r,z)\end{array}\right\}=\mathop{\underbrace{\left[\begin{array}{cc}{k}_{11} & {k}_{12}\\ {k}_{21} & {k}_{22}\end{array}\right]}}\limits_{{\rm{K}}}\,\left\{\begin{array}{c}{\widehat{\phi }}_{s}(r,z)\\ {\widehat{\phi }}_{f}(r,z)\end{array}\right\},$$

where

$$\begin{array}{ll}{k}_{11}=\frac{{\omega }^{2}(\rho -\alpha {\rho }_{f})}{M{\alpha }^{2}-2\mu -{\lambda }_{c}}, & {k}_{12}=\frac{{\omega }^{2}({\rho }_{f}-\alpha {\rho }_{m})}{M{\alpha }^{2}-2\mu -{\lambda }_{c}},\\ {k}_{21}=\frac{{\omega }^{2}((2\mu +{\lambda }_{c}){\rho }_{f}-M\alpha \rho )}{M(M{\alpha }^{2}-2\mu -{\lambda }_{c})}, & {k}_{22}=\frac{{\omega }^{2}((2\mu +{\lambda }_{c}){\rho }_{m}-M\alpha {\rho }_{f})}{M(M{\alpha }^{2}-2\mu -{\lambda }_{c})}.\end{array}$$

The K matrix can be rewritten using the Eigen decomposition method:

8 $$K=P\ D\ {P}^{-1},$$

where *P* is the eigenvector matrix and *D* is the eigenvalue matrix of the *K* matrix:

$$\begin{array}{rcl}P & = & \frac{1}{{k}_{21}}\left\{\begin{array}{cc}-\frac{\sqrt{{\left({k}_{11}-{k}_{22}\right)}^{2}+4{k}_{12}{k}_{21}}-{k}_{11}+{k}_{22}}{2} & \frac{\sqrt{{\left({k}_{11}-{k}_{22}\right)}^{2}+4{k}_{12}{k}_{21}}+{k}_{11}-{k}_{22}}{2}\\ {k}_{21} & {k}_{21}\end{array}\right\},\\ D & = & \left\{\begin{array}{cc}\frac{1}{2}\left(-\sqrt{{\left({k}_{11}-{k}_{22}\right)}^{2}+4{k}_{12}{k}_{21}}+{k}_{11}+{k}_{22}\right) & 0\\ 0 & \frac{1}{2}\left(\sqrt{{\left({k}_{11}-{k}_{22}\right)}^{2}+4{k}_{12}{k}_{21}}+{k}_{11}+{k}_{22}\right)\end{array}\right\}.\end{array}$$

It should be noted that Eq. (8) is still valid after neglecting the term $\frac{1}{{k}_{21}}$ in the eigenvector matrix *P* due to the existence of the term *P*^−1^. Introducing Eq. (8) into Eq. (7) and by multiplying *P*^−1^ and *P* in the left and right sides, respectively, we can obtain:

9 $${P}^{-1}\,{\nabla }^{2}\widehat{\overrightarrow{\phi }}(r,z)\,P=D\,{P}^{-1}\,\widehat{\overrightarrow{\phi }}(r,z)\,P.$$

By setting $\widehat{\overrightarrow{\phi }}(r,z)=P\,\overrightarrow{y}(r,z)$ in which $\overrightarrow{y}(r,z)=[{\widehat{\phi }}_{p1}(r,z),{\widehat{\phi }}_{p2}(r,z)]$, the system is finally decoupled as:

10 $${\nabla }^{2}\overrightarrow{y}(r,z)=D\overrightarrow{y}(r,z).$$

Under axisymmetric conditions, Eq. (10) for $\overrightarrow{y}(r,z)=[{\widehat{\phi }}_{p1}(r,z),{\widehat{\phi }}_{p2}(r,z)]$ in cylindrical coordinates is written as:

11a $$\left(\frac{{\partial }^{2}{\widehat{\phi }}_{p1}(r,z)}{\partial {r}^{2}}+\frac{1}{r}\frac{\partial {\widehat{\phi }}_{p1}(r,z)}{\partial r}+\frac{{\partial }^{2}{\widehat{\phi }}_{p1}(r,z)}{\partial {z}^{2}}\right)-{D}_{11}{\widehat{\phi }}_{p1}(r,z)=0,$$

11b $$\left(\frac{{\partial }^{2}{\widehat{\phi }}_{p2}(r,z)}{\partial {r}^{2}}+\frac{1}{r}\frac{\partial {\widehat{\phi }}_{p2}(r,z)}{\partial r}+\frac{{\partial }^{2}{\widehat{\phi }}_{p2}(r,z)}{\partial {z}^{2}}\right)-{D}_{22}{\widehat{\phi }}_{p2}(r,z)=0.$$

Since the variables ${\widehat{\phi }}_{p1}(r,z)$ and ${\widehat{\phi }}_{p2}(r,z)$ are a function of *r* and *z* in the cylindrical coordinates, the separation of variable $\,{\widehat{\phi }}_{p1}=\widehat{R}(r)\,\hat{Z}(z)$ can be used. By setting the both sides equal to  − *k*^2^ where *k* is the wavenumber in the radial direction, we can obtain the following equations:

12a $$\frac{{d}^{2}\widehat{R}(r)}{d{r}^{2}}+\frac{1}{r}\frac{d\widehat{R}(r)}{dr}+{k}^{2}\widehat{R}(r)=0,$$

12b $$\frac{{d}^{2}\hat{Z}(z)}{d{z}^{2}}-({k}^{2}+{D}_{11})\,\hat{Z}(z)=0.$$

The solutions to Eqs. (12a and 12b) are:

13a $$\widehat{R}(r)={C}_{1}\ {J}_{0}(kr),$$

13b $$\widehat{R}(z)={C}_{2}\ {e}^{-\sqrt{{k}^{2}+{D}_{11}}z},$$

in which *J*_0_ is the Bessel function of the first kind; *C*_1_ and *C*_2_ are the coefficients to be determined from the boundary conditions. Similarly, the solution for ${\widehat{\phi }}_{p1}$ can be obtained. The solution for $\overrightarrow{y}=[{\widehat{\phi }}_{p1},{\widehat{\phi }}_{p2}]$ is summarized as:

14a $${\widehat{\phi }}_{p1}(r,z)=A{e}^{-\sqrt{{k}^{2}+{D}_{11}}z}{J}_{0}(kr),$$

14b $${\widehat{\phi }}_{p2}(r,z)=B{e}^{-\sqrt{{k}^{2}+{D}_{22}}z}{J}_{0}(kr),$$

where *A* and *B* are the coefficients to be determined from the boundary conditions. For simplicity, the term $\sqrt{{k}^{2}+{D}_{11}}$ and $\sqrt{{k}^{2}+{D}_{22}}$ is denoted as *k*_*p*1_ and *k*_*p*2_, respectively.

Since $\widehat{\overrightarrow{\phi }}=P\overrightarrow{y}$, the solution for ${\widehat{\phi }}_{s}$ and ${\widehat{\phi }}_{f}$ can be finally obtained as:

15a $${\widehat{\phi }}_{s}(r,z)={p}_{11}A{e}^{-\sqrt{{k}^{2}+{D}_{11}}z}{J}_{0}(kr)+{p}_{12}B{e}^{-\sqrt{{k}^{2}+{D}_{22}}z}{J}_{0}(kr),$$

15b $${\widehat{\phi }}_{f}(r,z)={p}_{21}A{e}^{-\sqrt{{k}^{2}+{D}_{11}}z}{J}_{0}(kr)+{p}_{22}B{e}^{-\sqrt{{k}^{2}+{D}_{22}}z}{J}_{0}(kr).$$

### Solution of rotational wave (S wave)

The rotational wave is governed by Eqs. (5c) and (5d). By replacing ${\widehat{\overrightarrow{\psi }}}_{f}$ by ${\widehat{\overrightarrow{\psi }}}_{s}$, we obtain:

16 $${\nabla }^{2}{\widehat{\overrightarrow{\psi }}}_{s}(r,z)-\frac{\left(\frac{{\rho }_{f}^{2}}{{\rho }_{m}}-\rho \right){\omega }^{2}}{\mu }{\widehat{\overrightarrow{\psi }}}_{s}(r,z)=0.$$

Under axisymmetric conditions, the solution for Eq. (16) in the cylindrical coordinates is obtained as:

17 $${\widehat{\psi }}_{s}(r,z)=C{e}^{-\sqrt{{k}^{2}+\frac{\left(\frac{{\rho }_{f}^{2}}{{\rho }_{m}}-\rho \right){\omega }^{2}}{\mu }}z}{J}_{1}(k\ r),$$

where *C* is the coefficient to be determined from the boundary conditions and *J*_1_ is the Bessel function of the first kind of order one. For simplicity, the term $\sqrt{{k}^{2}+\frac{\left(\frac{{\rho }_{f}^{2}}{{\rho }_{m}}-\rho \right){\omega }^{2}}{\mu }}$ is denoted as *k*_*s*_.

### Displacement, stress and pore-water pressure in terms of potentials

In the cylindrical coordinates (*r*, *θ*, *z*), considering the axisymmetric conditions $\left(\frac{\partial }{\partial \theta }=0\right)$, the vector potential $\widehat{\overrightarrow{\psi }}$ has only the component in the *θ* direction that does not vanish. For simplicity, the vector potential $\widehat{\overrightarrow{\psi }}$ in the *θ* direction is denoted as ${\widehat{\phi }}_{s}$ and ${\widehat{\phi }}_{f}$ for solid skeleton and porewater, respectively. This property reduces the displacement to the following forms:

18a $${{\hat{u}}}_{r}(r,z)=\frac{\partial {\widehat{\phi }}_{s}(r,z)}{\partial r}-\frac{\partial {\widehat{\psi }}_{s}(r,z)}{\partial z},\ {{\hat{u}}}_{z}(r,z)=\frac{\partial {\widehat{\phi }}_{s}(r,z)}{\partial z}+\frac{1}{r}\frac{\partial (r{\widehat{\psi }}_{s}(r,z))}{\partial r},$$

18b $${\hat{w}}_{r}(r,z)=\frac{\partial {\widehat{\phi }}_{f}(r,z)}{\partial r}-\frac{\partial {\widehat{\psi }}_{f}(r,z)}{\partial z},\ {\hat{w}}_{z}(r,z)=\frac{\partial {\widehat{\phi }}_{f}(r,z)}{\partial z}+\frac{1}{r}\frac{\partial (r{\widehat{\psi }}_{f}(r,z))}{\partial r}.$$

The effective stress and pore-water pressure are written as:

19a $${\widehat{\sigma }}_{zr}^{{\prime} }(r,z)=\mu \left(\frac{\partial {{\hat{u}}}_{r}(r,z)}{\partial z}+\frac{\partial {{\hat{u}}}_{z}(r,z)}{\partial r}\right),$$

19b $${\widehat{\sigma }}_{zz}^{{\prime} }(r,z)=\lambda \left(\frac{\partial {{\hat{u}}}_{r}(r,z)}{\partial r}+\frac{{{\hat{u}}}_{r}(r,z)}{r}+\frac{\partial {{\hat{u}}}_{z}(r,z)}{\partial z}\right)+2\mu \frac{\partial {{\hat{u}}}_{z}(r,z)}{\partial z},$$

19c $$\widehat{p}(r,z)=-\alpha M\left(\frac{{\partial }^{2}{\widehat{\phi }}_{s}(r,z)}{\partial {r}^{2}}+\frac{1}{r}\frac{\partial {\widehat{\phi }}_{s}(r,z)}{\partial r}+\frac{{\partial }^{2}{\widehat{\phi }}_{s}(r,z)}{\partial {z}^{2}}\right)-M\left(\frac{{\partial }^{2}{\widehat{\phi }}_{f}(r,z)}{\partial {r}^{2}}+\frac{1}{r}\frac{\partial {\widehat{\phi }}_{f}(r,z)}{\partial r}+\frac{{\partial }^{2}{\widehat{\phi }}_{f}(r,z)}{\partial {z}^{2}}\right).$$

### Spectral element formulation for dynamic poroelasticity

In u-w formulation (displacement of solid and relative displacement of porewater), the displacement components *w*_*r*_ and *w*_*z*_ are linearly dependent. In this paper, only *w*_*z*_ is used in the stiffness matrix. For two-node elements where a layer has a finite thickness, the matrix for the displacement components are written as follows:

20 $$\left[\begin{array}{c}{{\hat{u}}}_{r1}(r,z)\\ {{\hat{u}}}_{z1}(r,z)\\ {\hat{w}}_{z1}(r,z)\\ {{\hat{u}}}_{r2}(r,z)\\ {{\hat{u}}}_{z2}(r,z)\\ {\hat{w}}_{z2}(r,z)\end{array}\right]=\mathop{\underbrace{\left[\begin{array}{cccccc}-k{p}_{11} & -k{p}_{12} & {k}_{s} & -{e}^{-h{k}_{p1}}k{p}_{11} & -{e}^{-h{k}_{p2}}k{p}_{12} & -{e}^{-h{k}_{s}}{k}_{s}\\ -{k}_{p1}{p}_{11} & -{k}_{p2}{p}_{12} & k & {e}^{-h{k}_{p1}}{k}_{p1}{p}_{11} & {e}^{-h{k}_{p2}}{k}_{p2}{p}_{12} & {e}^{-h{k}_{s}}k\\ -{k}_{p1}{p}_{21} & -{k}_{p2}{p}_{22} & -\frac{{\rho }_{f}}{{\rho }_{m}}k & {e}^{-h{k}_{p1}}{k}_{p1}{p}_{21} & {e}^{-h{k}_{p2}}{k}_{p2}{p}_{22} & -\frac{{\rho }_{f}}{{\rho }_{m}}{e}^{-h{k}_{s}}k\\ -{e}^{-h{k}_{p1}}k{p}_{11} & -{e}^{-h{k}_{p2}}k{p}_{12} & {e}^{-h{k}_{s}}{k}_{s} & -k{p}_{11} & -k{p}_{12} & -{k}_{s}\\ -{e}^{-h{k}_{p1}}{k}_{p1}{p}_{11} & -{e}^{-h{k}_{p2}}{k}_{p2}{p}_{12} & {e}^{-h{k}_{s}}k & {k}_{p1}{p}_{11} & {k}_{p2}{p}_{12} & k\\ -{e}^{-h{k}_{p1}}{k}_{p1}{p}_{21} & -{e}^{-h{k}_{p2}}{k}_{p2}{p}_{22} & -\frac{{\rho }_{f}}{{\rho }_{m}}{e}^{-h{k}_{s}}k & {k}_{p1}{p}_{21} & {k}_{p2}{p}_{22} & -\frac{{\rho }_{f}}{{\rho }_{m}}k\end{array}\right]}}\limits_{{S}_{1}^{{\prime} }}\,\left[\begin{array}{c}{A}_{1}\\ {B}_{1}\\ {C}_{1}\\ {A}_{2}\\ {B}_{2}\\ {C}_{2}\end{array}\right].$$

Similarly, the matrix for effective stress components and porewater pressure in frequency domain is shown in Eq. 21 in which the components for matrix ${S}_{2}^{{\prime} }$ can be found in Appendix A.

21 $$\left[\begin{array}{c}{\widehat{\sigma }}_{zr1}^{{\prime} }(r,z)\\ {\widehat{\sigma }}_{zz1}^{{\prime} }(r,z)\\ {\widehat{p}}_{1}(r,z)\\ {\widehat{\sigma }}_{zr2}^{{\prime} }(r,z)\\ {\widehat{\sigma }}_{zz2}^{{\prime} }(r,z)\\ {\widehat{p}}_{2}(r,z)\end{array}\right]=\mathop{\underbrace{\left[\begin{array}{cccccc}{m}_{11} & {m}_{12} & {m}_{13} & {m}_{14} & {m}_{15} & {m}_{16}\\ {m}_{21} & {m}_{22} & {m}_{23} & {m}_{24} & {m}_{25} & {m}_{26}\\ {m}_{31} & {m}_{32} & {m}_{33} & {m}_{34} & {m}_{35} & {m}_{36}\\ {m}_{41} & {m}_{42} & {m}_{43} & {m}_{44} & {m}_{45} & {m}_{46}\\ {m}_{51} & {m}_{52} & {m}_{53} & {m}_{54} & {m}_{55} & {m}_{56}\\ {m}_{61} & {m}_{62} & {m}_{63} & {m}_{64} & {m}_{65} & {m}_{66}\end{array}\right]}}\limits_{\begin{array}{c}{S}_{2}^{{\prime} }\end{array}}\,\left[\begin{array}{c}{A}_{1}\\ {B}_{1}\\ {C}_{1}\\ {A}_{2}\\ {B}_{2}\\ {C}_{2}\end{array}\right].$$

According to the Cauchy stress principle, the traction force (${[{\bar{T}}_{rz1},{\bar{T}}_{z1},{\bar{T}}_{1}{\bar{T}}_{rz2}.{\bar{T}}_{z2},{\bar{T}}_{2}]}^{T}$) is taken as the dot product between the stress tensor and the unit vector along the outward normal direction. Due to the convention that the upward direction is negative, the upper boundary becomes (${[-{\widehat{\sigma }}_{rz1},-{\widehat{\sigma }}_{zz1},-{\widehat{p}}_{1}]}^{T}$). Similarly, to make the sign consistent, the *N* matrix is applied to matrix ${S}_{2}^{{\prime} }\ \cdot \ {S}_{1}^{{}^{{\prime} }-1}$. In the future, the matrix $N\cdot {S}_{2}^{{\prime} }\ \cdot \ {S}_{1}^{{}^{{\prime} }-1}$ will be denoted as the *G*_*i*_ matrix, in which *i* denotes the layer number.

22 $${\left\{\begin{array}{c}{\bar{T}}_{rz1}\\ {\bar{T}}_{z1}\\ {\bar{T}}_{1}\\ {\bar{T}}_{rz2}\\ {\bar{T}}_{z2}\\ {\bar{T}}_{2}\end{array}\right\}}_{i}={\left\{\begin{array}{c}-{\widehat{\sigma }}_{rz1}(r,z)\\ -{\widehat{\sigma }}_{zz1}(r,z)\\ -{\widehat{p}}_{1}(r,z)\\ {\widehat{\sigma }}_{rz2}(r,z)\\ {\widehat{\sigma }}_{zz2}(r,z)\\ {\widehat{p}}_{2}(r,z)\end{array}\right\}}_{i}=\mathop{\underbrace{N\cdot {S}_{2}^{{\prime} }\cdot {S}_{1}^{{}^{{\prime} }-1}}}\limits_{{G}_{i}}\cdot {\left\{\begin{array}{c}{{\hat{u}}}_{r1}(r,z)\\ {{\hat{u}}}_{z1}(r,z)\\ {\hat{w}}_{z1}(r,z)\\ {{\hat{u}}}_{r2}(r,z)\\ {{\hat{u}}}_{z2}(r,z)\\ {\hat{w}}_{z2}{(r,z)}_{i},\end{array}\right\}}_{i},$$

where

23 $$N=\left[\begin{array}{cccccc}-1 & 0 & 0 & 0 & 0 & 0\\ 0 & -1 & 0 & 0 & 0 & 0\\ 0 & 0 & -1 & 0 & 0 & 0\\ 0 & 0 & 0 & 1 & 0 & 0\\ 0 & 0 & 0 & 0 & 1 & 0\\ 0 & 0 & 0 & 0 & 0 & 1\end{array}\right].$$

**Figure 2:**
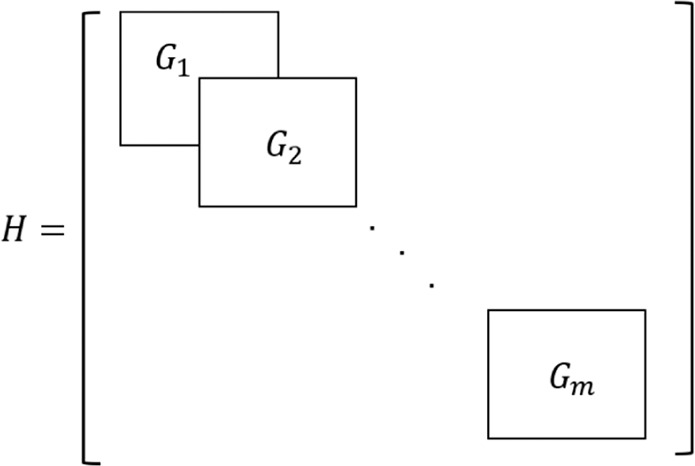
Global stiffness matrix construction.

After obtaining the stiffness matrix for each element, the global stiffness matrix can be obtained by applying the continuity conditions between the layer interfaces. The stiffness assembling method is shown in Fig. 2. The global stiffness is denoted as *H* matrix for simplicity. An example of the global stiffness matrix for a two layer system is provided in Appendix B.

### Soil response under dynamic load (boundary conditions)

In the ultrasonic tests, a vertical impulse load *f*(*t*, *r*) is applied to one end of the soil specimen. The surface is assumed to be permeable, which implies the porewater pressure at the surface is zero. Under such conditions, the displacements in the frequency domain can be written as:

24 $$\left\{\begin{array}{c}0\\ \widehat{f}(s,r)\\ 0\\ \cdot \\ \cdot \\ \cdot \\ 0\end{array}\right\}=\left\{\begin{array}{ccccccc} &  &  &  &  &  & \\  &  &  &  &  &  & \\  &  &  &  &  &  & \\  &  &  & H &  &  & \\  &  &  &  &  &  & \\  &  &  &  &  &  & \\  &  &  &  &  &  & \end{array}\right\}\left\{\begin{array}{c}{{\hat{u}}}_{r1}\\ {{\hat{u}}}_{z1}\\ {\hat{w}}_{z1}\\ \cdot \\ \cdot \\ \cdot \\ {\hat{w}}_{zn}\end{array}\right\}.$$

The impulse load *f* is firstly defined in time domain and can decomposed into two independent functions in terms of time variable *f*_*n*_(*t*) and radial variable *f*_*r*_(*r*):

25 $$f(t,r)={f}_{n}(t)\ {f}_{r}(r).$$

The mathematical expression for the function *f*_*n*_(*t*) depends mainly on the type of impulse loads created by the signal generator. In this paper, a sinusoidal impulse function is used as the external load to simulate the applied load. The load with amplitude of one is mathematically described in Eq. (26).

26 $${f}_{n}(t)=sin(2\pi ft)\ [1-H(t-1/f)],$$

where *t*(*s*) is time and *f*(*Hz*) is the frequency; *H*() is the Heaviside step function.

Meanwhile, the function *f*_*r*_(*r*) is normally written using the Fourier-Bessel series:

27 $${f}_{r}(r)=\mathop{\sum }\limits_{m=1}^{\infty }{F}_{m}{J}_{0}({k}_{m}r),$$

where

$${F}_{m}(m)=\frac{2{r}_{0}\sin \left({r}_{0}{k}_{m}\right)}{{r}_{\infty }^{2}{k}_{m}{J}_{1}^{2}\left({r}_{\infty }{k}_{m}\right)}\frac{n\,+\,1-m}{n+1},$$

where *r*_0_ is the radius of the contact area; *k*_*m*_ is the mode number; *n* is the total mode number; *r*_*∞*_ is the diameter of the soil specimen.

The displacement obtained in Eq. (24) is in the frequency domain. To obtain the soil response in time domain, the numerical Durbin inverse transform method is applied^23^:

28 $${{\mathcal{L}}}^{-1}\{\widehat{\theta }(s)\}=\theta (t)={\int }_{0}^{\infty }\widehat{\theta }(s){e}^{st}ds.$$

## Results and Discussion

The characterization of porosity has been a challenge because soil porosity can not be captured through traditional low-frequency tests. Such limitations can be explained by comparing the size of pore space and wavelength. A sensitivity analysis of the soil porosity is performed to verify such limitations. In this study, a soil column with a height and radius of 0.1 m is studied. The impulse load is applied to an area with a radius of 1*cm* at the center of the top end of the soil column. The displacement at the center (*r* = 0) in the other end is recorded and compared.

The typical values of Young’s modulus, porosity, density, permeability and Poisson’s ratio are well documented in the literature^24–27^. For example, high-plasticity clay (CH based on the Unified Soil Classification System (USCS)) has a Young’s modulus ranging from 0.35 to 32 MPa and porosity from 0.39 to 0.59; Silts and clays of low plasticity (ML, CL) have a typical value of Young’s modulus ranging from 1.5 to 60 MPa and porosity from 0.29 to 0.56; poorly graded sands (SP) normally have a Young’s modulus from 10 to 80 MPa and porosity from 0.23 to 0.43; The Young’s modulus of well-graded gravel (GW) is between 30-320 MPa and its porosity is from 0.21 to 0.32. The average dry density ranges from 1700 to 2300 *kg*∕*m*^3^. The average permeability varies from 5 × 10^−10^ (clay of high plasticity) to 0.4 m/s (sand and gravel). The typical values of Poisson’s ratio vary from 0.1 to 0.49 for clay and from 0.3 to 0.35 for silt.

In this paper, two groups of soils are studied: the first group includes clay, silt, sand and loose gravel which generally have a relatively low Young’s modulus (lower than 100 MPa). The second group includes dense gravel which has a Young’s modulus equal or greater than 200 MPa.

### The effect of frequency and soil parameters on dynamic response

**Figure 3:**
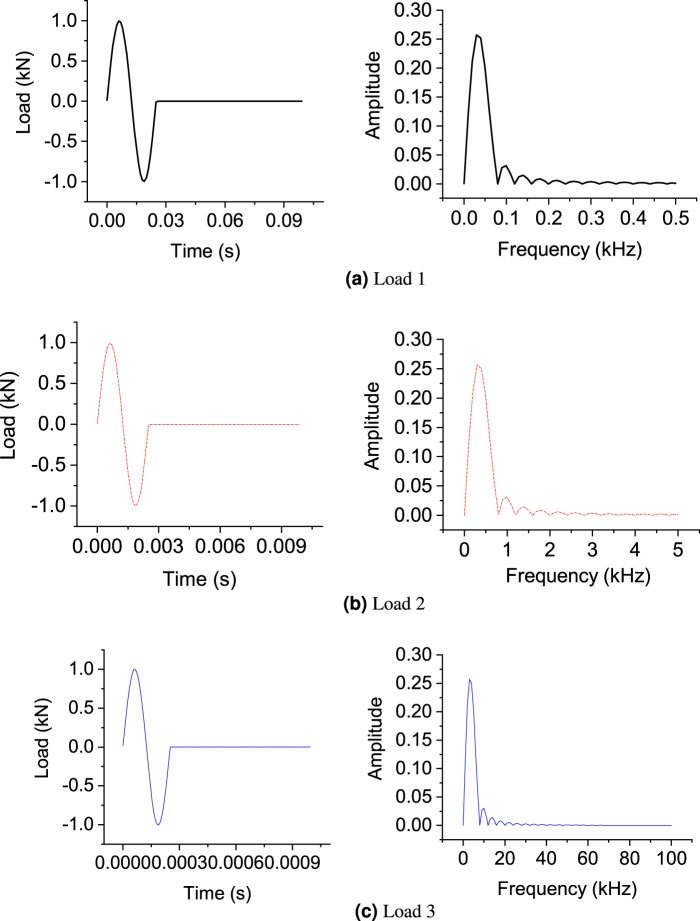
Impulse load in time and frequency domains.

**Figure 4:**
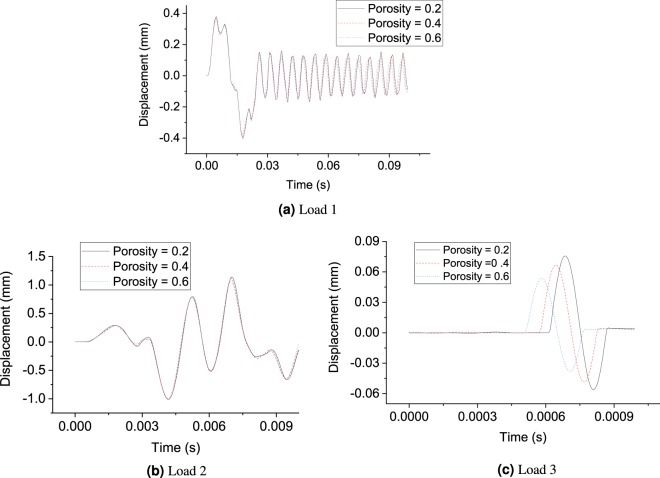
Sensitivity analysis of porosity under (**a**) load 1 (**b**) load 2 and (**c**) load 3.

The effect of impulse load frequency and soil parameters on the dynamic soil response is studied in this section for the above-mentioned groups of soils. For the first group, the soil properties are taken as: Young’s modulus is 20 MPa; Poisson’s ratio is 0.35; dry density is 1800 *kg*∕*m*^3^. The wavelength can be calculated using the algorithm shown in Appendix C. Several sensitivity analyses under three impulse loads with various predominant frequencies are performed. The impulse load distributions in time and frequency domains are shown in Fig. 3. The loads 1, 2 and 3 have a predominant frequency of 0.05, 0.5 and 5 kHz, respectively. The amplitude of the input force is assumed to be 1 kN. The corresponding soil response at the receiver location is shown in Fig. 4.

As shown in Fig. 4, the different porosities (0.2, 0.4 and 0.6) give similar output displacement for load 1 and 2, which verifies that the size of pore space is not captured by the low-frequency impulse loads. In the inversion process, the porosity will be located at the shallow dimension, which makes the optimization algorithm difficult to be updated. Therefore, the characterization of saturated soil under low-frequency impulse load (below 5 kHz in this case) is nearly impossible. However, in the case of load 3 with a predominant frequency around 5 kHz, the effect of porosity is clearly triggered. The pore-scale of sand, for example, is around 760 *μ*m as reported by^28^. Through the root search algorithm described in Appendix C, the wavelength under the load 3 is calculated around 1000–2000 *μ*m, which is close to the poro-space scale of the studied soil. Therefore, the impulse load 3 is a good choice for the lab-scale characterization of soil specimens for group 1.

**Figure 5:**
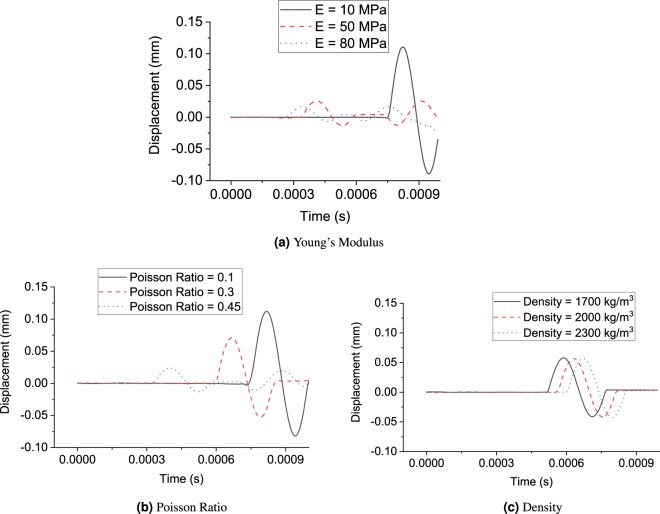
Sensitivity analysis of soil (group 1) parameters under impulse load.

Similarly, the sensitivity analyses are performed by considering different densities, Young’s modulus and Poisson’s ratios. The output displacement is shown in Fig. 5. The effects of Young’s modulus, Poisson’s ratio and density of soil are also shown in Fig. 5. A higher Young’s modulus leads to a faster wave travelling speed and a smaller amplitude of the output wave. A higher density, on the contrary, leads to a lower travelling wave speed. Poisson’s ratio that measures the tendency of material to expand in directions perpendicular to the direction of compression has an inverse relation with the wave speed. Therefore, it can be seen that the distribution of the output stress wave is a function of porosity, density, Young’s modulus and Poisson’s ratio.

**Figure 6:**
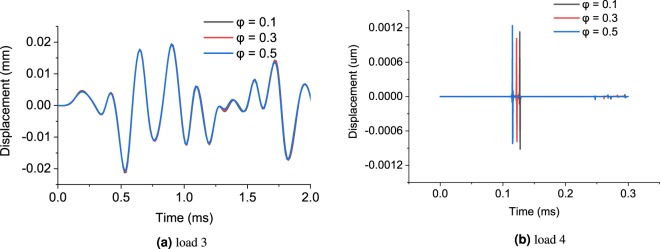
Sensitivity of soil parameters under impulse load for dense gravel.

**Figure 7:**
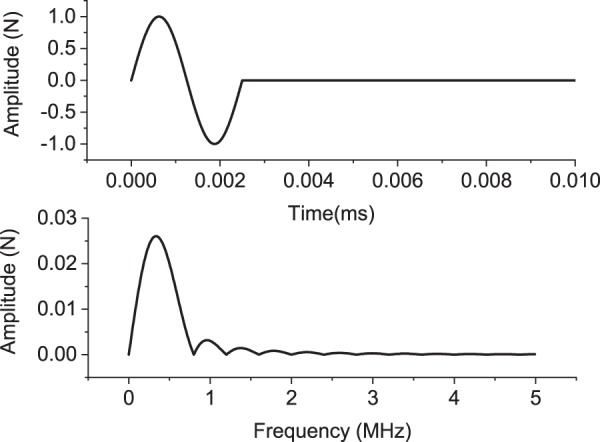
High-frequency (ultrasonic) impulse load 4 in time and frequency domain.

In the case of soil group 2, dense gravel whose Young’s modulus is up to 320 MPa, it is found that the load 3 (up to 5kHz) generates similar displacement outputs at different porosities (0.1, 0.3 and 0.5), as shown in Fig. 6. It means that load 3 can not trigger the effect of porosity. In order to characterize the porosity for very dense soils, one of the techniques is to further reduce the wavelength of the stress wave by increasing the frequency of the impulse load. It is found that an impulse load 4 with a higher predominant frequency (up to 0.5 MHz), as shown in Fig. 7, can effectively differentiate dense soils with various porosities.

### Case study

In this section, a case study is presented to show the process of saturated soil characterization. For this purpose, a synthetic data is firstly generated to simulate real measurements. For simplicity, the results are only presented for soil group 1. The nature of this inversion problem and inversion algorithm selection are discussed in detail in the following sections. At the end, the inversion results (soil parameters) are given based on the synthetic data and selected inversion algorithm.

#### Synthetic data

**Figure 8:**
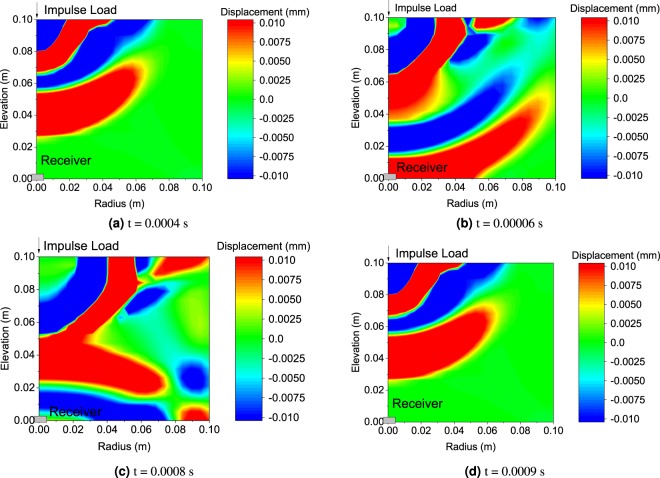
Displacement contour snap shots at various time.

A synthetic data (the displacement measured by a piezoelectric receiver) is firstly obtained using the following settings: Young’s modulus is 20 MPa; Poisson’s ratio is 0.35; density of solid skeleton is 1800 *kg*/*m*^3^ and porosity is taken as 0.3; The time interval is set to be 2 ms. Under the impulse load 3, as shown in Fig. 3, the snap shot of displacement contours (symmetric) at various time spans are shown in Fig. 8. The locations of impulse load and receiver are shown in Fig. 8. It is shown that the stress wave propagates through the sample and reaches the receiver at about 0.6 ms. The wave reflection at the bottom boundary is clearly visualized at time 0.8 ms and 0.9 ms.

**Figure 9:**
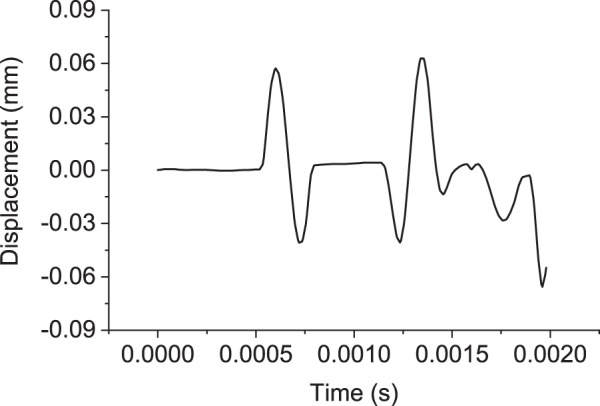
Soil dynamic response measured at the receiver location under impulse load 3.

The response measured at the receiver location is summarised in Fig. 9. In the laboratory ultrasonic test, the soil response is only recorded at the receiver location. Thus, in the following inversion process, only the results at the receiver location will be used as the input instead of the displacement at the entire domain.

#### Inversion algorithm

The inversion algorithm takes the measured displacement at the receiver location (shown in Fig. 9) as the input. The goal of the inversion process is to predict the soil properties including Young’s modulus, Poisson’s ratio, density and porosity based on the receiver signals. Given the initial guesses for the soil parameters, the inversion algorithm updates the prediction based on the difference between the displacement measured by the receiver and the predicted displacement response.

The update process can be achieved through the gradient-based and gradient-free optimization method. The gradient-based optimization is efficient in large convex problems such as linear least square problems and are commonly used in large optimization problems (e.g. deep learning and adjoint method). Therefore, the gradient based method is preferred in most cases, especially for convex optimization problems. However, such a method is highly likely to be affected by the local minimum since the gradient at any local minimum is zero. Thus, it is not favorable for non-convex problems.

**Figure 10:**
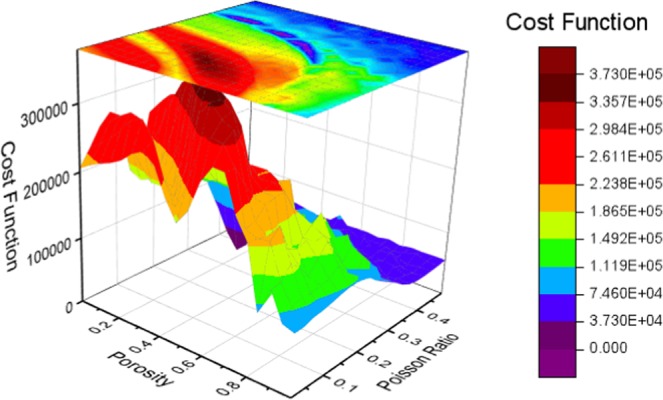
Non-convex optimization space for porosity and Poisson’s ratio.

An analysis was performed to show the nature of the soil characterization optimization problem. It is important to determine whether such application belongs to convex or non-convex problem. Then the corresponding optimization algorithm can be selected based on the nature of the problem. The aim (cost) function is defined as the Euclidean norm between the synthetic and predicted data. The optimization space can be visualized by performing parameter sweep. For example, the optimization space for the porosity and Poisson’s ratio is shown in Fig. 10.

It is shown in Fig. 10 that a multiple local minimum exists in the optimization space. Therefore, the characterization of soil parameters is a non-convex optimization problem. If the gradient-based optimization algorithm is applied, the predictions will be highly dependent on the initial guess, which may leads to erroneous predictions in most cases. To make the estimation robust and accurate, a global optimization algorithm is favorable. In this work, the differential evolution algorithm that is designed for nonlinear and non-differential problems is used. Such an algorithm requires fewer control variables in comparison to other algorithms (e.g. genetic algorithm) and can be easily implemented in parallel computation^29^.

**Figure 11:**
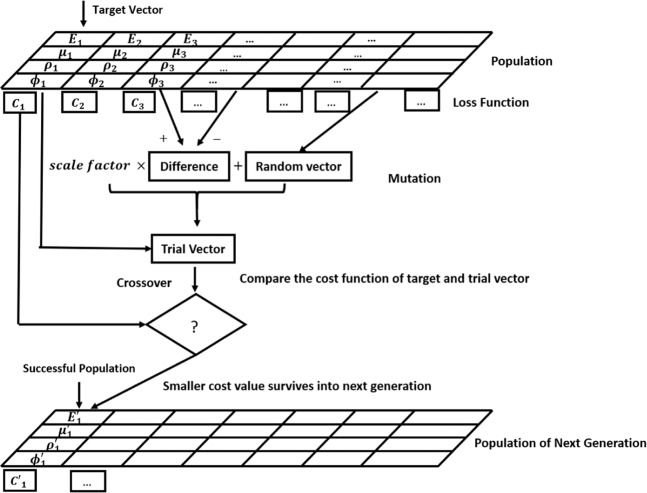
Flowchart of differential evolution for the optimization of soil parameters.

A brief description of the differential evolution algorithm is given in Fig. 11. First, a population of candidate solutions are generated randomly; Then by moving around in the search space through a combination of the existing temporary solutions, a series of better solutions is expected to be obtained. In the differential evolution, the mutation constant is taken in the range of 0.5 to 1 and the recombination constant is recommended to be 0.9^30^.

#### Inversion results

**Figure 12:**
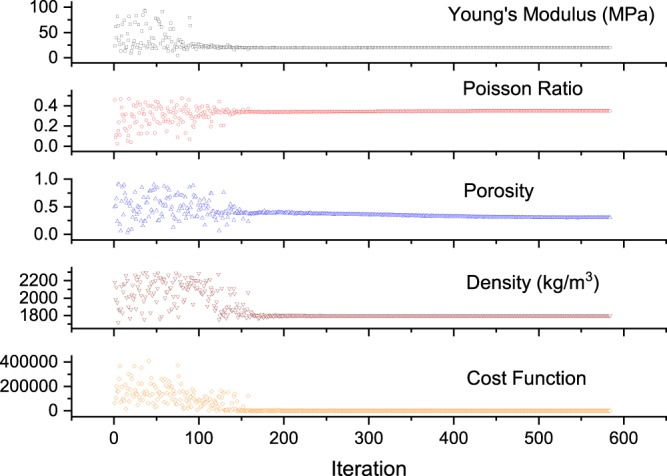
Updates of each parameter through a differential evolution algorithm.

Combining the synthetic data (as the input) shown in Fig. 9 and the differential evolution algorithm described above, the updates of the soil parameters and the corresponding values of the cost function are shown in Fig. 12. The iteration number shows the number of times that the forward problem is solved independently. After 200 iterations, the differential evolution algorithm stabilizes. The predicted soil parameters are as follows: Young’s modulus is 20 MPa; Poisson’s ratio is 0.35; density is 1800 *kg*∕*m*^3^; porosity is 0.3 and loss function is 0. It can be seen that the prediction of soil parameters based on the transmitted wave measured by the receiver (as shown in Fig. 9) is exactly the same as the original input.

**Figure 13:**
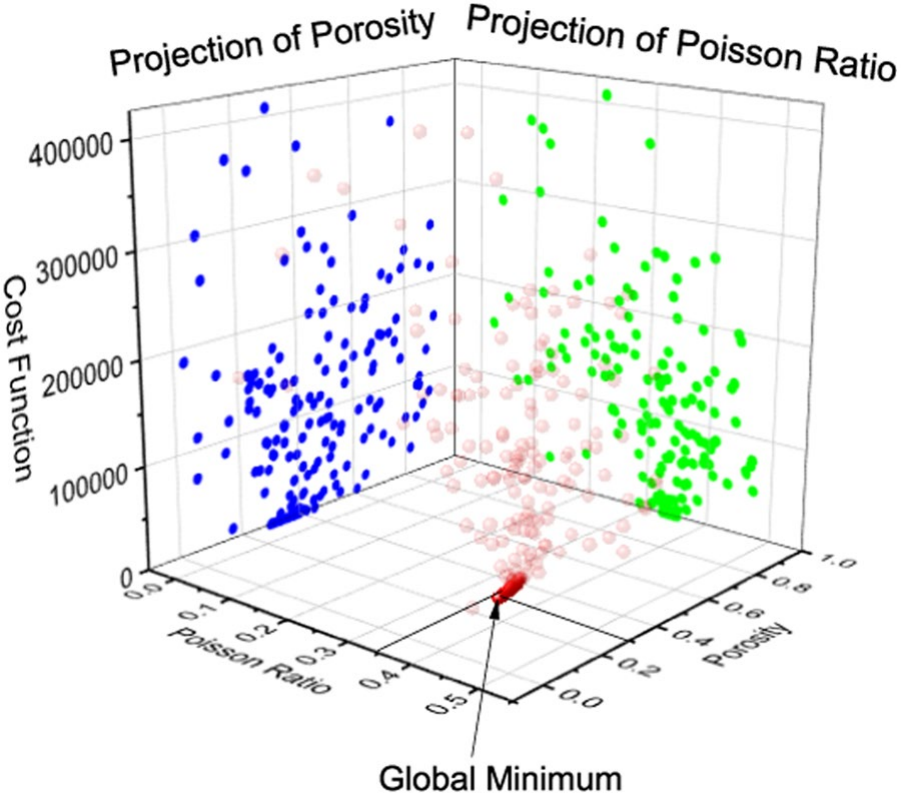
Updates of Poisson’s ratio and porosity through a differential evolution algorithm.

**Figure 14:**
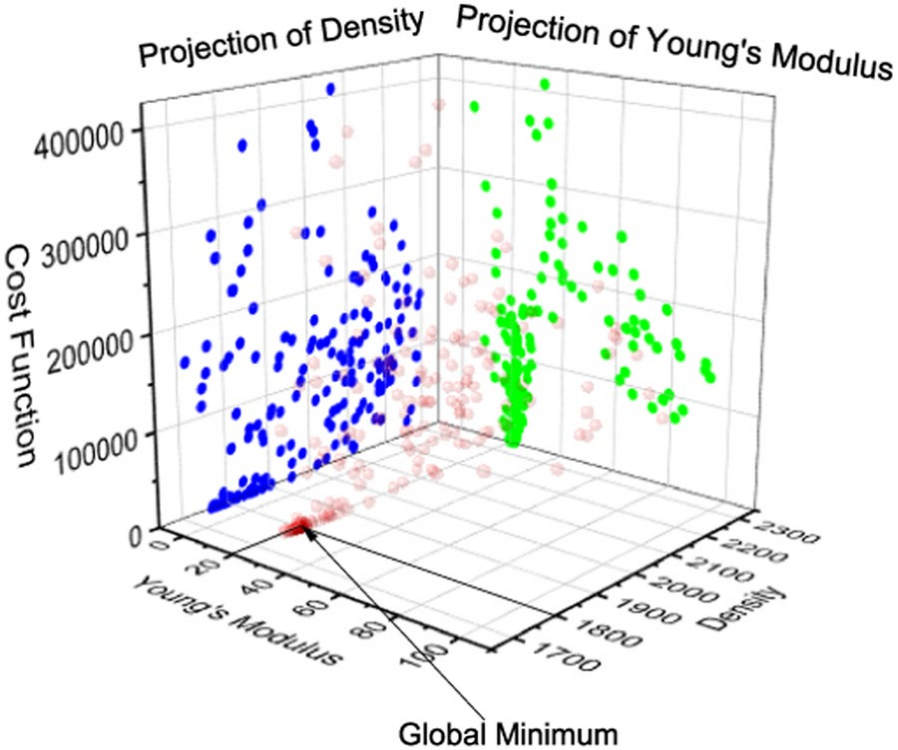
Updates of Young’s modulus and density through a differential evolution algorithm.

The differential evolution algorithm successfully finds the global minimum, despite of the existence of multiple local minimum. The spatial distribution of soil parameters updates are shown in Figs. 13 and 14. Through the projection of each parameter, it can be seen that Young’s modulus is relatively easier to update. For the other three parameters (Poisson’s ratio, density and porosity), there are multiple locations where cost function is close to zero. Thus, it took more number of iterations to update to the true values. However, it can be seen such a multidimensional optimization problem is well handled by the differential evolution algorithm.

### Uncertainty analysis

**Figure 15:**
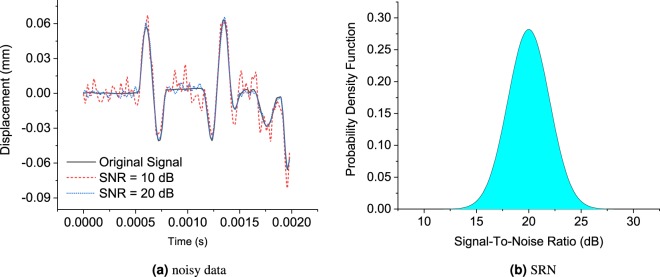
Probability density function for the signal to noise ratio.

The predicted soil properties (Young’s modulus, Poisson’s ratio, density and porosity) are likely to be affected by the noise level of the measurement data, which could be introduced by the sensor measurement errors and ambient noise. In this uncertainty analysis, random white noise is added to measured displacement data with targeted signal-to-noise (SRN) ratio. For example, the noisy data with 10 and 20 dB of SRN is shown in Fig. 15a. A normal distributed probability density function of SRN is used as the input to account the uncertainty introduced by noise, as shown in Fig. 15b. It is assumed that there is a 28% possibility to have a SRN of 20 dB in measured data.

**Figure 16:**
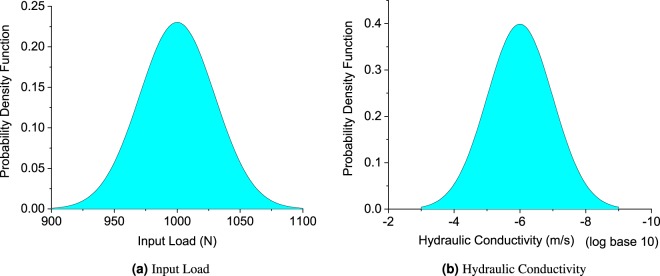
Probability density function for input load and hydraulic conductivity.

In addition, the uncertainty can be introduced by the unknown coupling performance in the interface of piezoelectric sensors and soil specimens. The input electricity signal does not necessarily generate the desired input pressure. To account for such uncertainties, the magnitude of input load is assumed to be in normal distribution, as shown in Fig. 16a. The uncertainty also comes from the inherent soil property assumptions made in soil specimen during the inversion analysis, such as hydraulic conductivity. Thus, a normal probability distribution is also applied to account such uncertainty, as shown in Fig. 16b.

**Figure 17:**
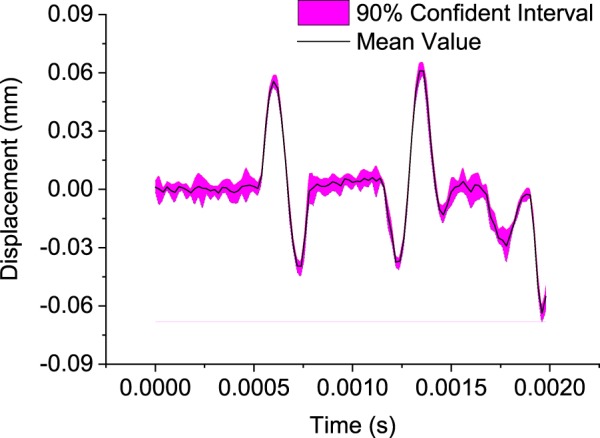
The 90% confidence interval of displacement distribution.

The generalized Polynomial Chaos Expansions (PCE) method developed by^31^ is used for the uncertainty analysis in this paper. The PCE technique, as a rigorous uncertainty quantification method, provides reliable numerical estimates of uncertain physical quantities. It was also reported that the PCE is much faster than Monte Carlo methods when the number of uncertainty parameters are lower than 20^32^. The 90% confident interval of the displacement at the receiver location is calculated through the PCE technique, shown in Fig. 17.

Then, based on the inversion analysis, the predicted soil properties in the 90% confidence interval are shown in Table 1. Then, the variation ratio is calculated by comparing the mean values (obtained through uncertainty analysis) with the original predictions. It is found the prediction of porosity could be affected by the uncertainty introduced by the white Gaussian noise, coupling effect between transmitter and soil specimen as well as other factors. However, various signal processing methods can be used to improve the noisy measurements.

**Table 1:** The soil parameter variation range based on uncertainty analysis.

Soil Properties	Lower Bound	Higher Bound	Variation Ratio
Young’s Modulus (MPa)	20.42	20.92	3.3%
Poisson Ratio	0.352	0.354	0.3%
Density (kg/m^3^)	1813.59	1878.58	2.6%
Porosity	0.26	0.27	11.7%

## Conclusions

In this paper, an ultrasonic-based characterization of soil specimens is developed for the instant measurement of soil properties including Young’s modulus and Poisson’s ratio (compression/shear wave velocity), density and porosity. The developed meshless semi-analytical algorithm reduces the computational effort significantly in comparison to standard numerical techniques such as the finite element method. In fact, the advantage of such a solution is that the dynamic response is evaluated at the receiver location only rather than the entire domain. The soil response in other locations is not measured in the real application and does not factor in soil characterization.

It is concluded that high-frequency impulse loads (with predominant frequency of up to 5 kHz) is required to trigger the effect of porosity for soils with relatively low Young’s modulus (e.g clay, silt and sand). For stiffer materials, such as very dense gravels, an impulse load with predominant frequency of 0.5 MHz is required to characterize their porous nature. The characterization of soil properties has been proved as a highly non-convex optimization problem in this paper. The differential evolution algorithm, as a global optimization method, is found efficient and effective in finding the optimum soil properties, such that the difference between the predicted and measured stress waves is minimized. In conclusion, the developed method in interpreting dynamic response of saturated soil can be used for the immediate characterization of Young’s modulus, Poisson’s ratio, density and porosity for a given soil specimen.

## Appendix A: Components of Matrix ${S}_{2}^{{\prime} }$

The components of the matrix ${S}_{2}^{{\prime} }$ for effective stress components and porewater pressure in frequency domain is shown as follows:

$$\begin{array}{lll}{m}_{11}=2k{k}_{p1}{p}_{11}\mu  & {m}_{12}=2k{k}_{p2}{p}_{12}\mu  & {m}_{13}=-({k}^{2}+{k}_{s}^{2})\mu \\ {m}_{14}=-2{e}^{-h{k}_{p1}}k{k}_{p1}{p}_{11}\mu  & {m}_{15}=-2{e}^{-h{k}_{p2}}k{k}_{p2}{p}_{12}\mu  & {m}_{16}=-{e}^{-h{k}_{s}}({k}^{2}+{k}_{s}^{2})\mu \\ {m}_{21}={p}_{11}({k}_{p1}^{2}(\lambda +2\mu )-{k}^{2}\lambda ) & {m}_{22}={p}_{12}({k}_{p2}^{2}(\lambda +2\mu )-{k}^{2}\lambda ) & {m}_{23}=-2k{k}_{s}\mu \\ {m}_{24}={e}^{-h{k}_{p1}}{p}_{11}({k}_{p1}^{2}(\lambda +2\mu )-{k}^{2}\lambda ) & {m}_{25}={e}^{-h{k}_{p2}}{p}_{12}({k}_{p2}^{2}(\lambda +2\mu )-{k}^{2}\lambda ) & {m}_{26}=2{e}^{-h{k}_{s}}k{k}_{s}\mu \\ {m}_{31}=(k-{k}_{p1})(k+{k}_{p1})M({p}_{21}+{p}_{11}\alpha ) & {m}_{32}=(k-{k}_{p2})(k+{k}_{p2})M({p}_{22}+{p}_{12}\alpha ) & {m}_{33}=0\\ {m}_{34}={e}^{-h{k}_{p1}}(k-{k}_{p1})(k+{k}_{p1})M({p}_{21}+{p}_{11}\alpha ) & {m}_{35}={e}^{-h{k}_{p2}}(k-{k}_{p2})(k+{k}_{p2})M({p}_{22}+{p}_{12}\alpha ) & {m}_{36}=0\\ {m}_{41}=2{e}^{-h{k}_{p1}}k{k}_{p1}{p}_{11}\mu  & {m}_{42}=2{e}^{-h{k}_{p2}}k{k}_{p2}{p}_{12}\mu  & {m}_{43}=-{e}^{-h{k}_{s}}({k}^{2}+{k}_{s}^{2})\mu \\ {m}_{44}=-2k{k}_{p1}{p}_{11}\mu  & {m}_{45}=-2k{k}_{p2}{p}_{12}\mu  & {m}_{46}=-({k}^{2}+{k}_{s}^{2})\mu \\ {m}_{51}={e}^{-h{k}_{p1}}{p}_{11}({k}_{p1}^{2}(\lambda +2\mu )-{k}^{2}\lambda ) & {m}_{52}={e}^{-h{k}_{p2}}{p}_{12}({k}_{p2}^{2}(\lambda +2\mu )-{k}^{2}\lambda ) & {m}_{53}=-2{e}^{-h{k}_{s}}k{k}_{s}\mu \\ {m}_{54}={p}_{11}({k}_{p1}^{2}(\lambda +2\mu )-{k}^{2}\lambda ) & {m}_{55}={p}_{12}({k}_{p2}^{2}(\lambda +2\mu )-{k}^{2}\lambda ) & {m}_{56}=2k{k}_{s}\mu \\ {m}_{61}={e}^{-h{k}_{p1}}(k-{k}_{p1})(k+{k}_{p1})M({p}_{21}+{p}_{11}\alpha ) & {m}_{62}={e}^{-h{k}_{p2}}(k-{k}_{p2})(k+{k}_{p2})M({p}_{22}+{p}_{12}\alpha ) & {m}_{63}=0\\ {m}_{64}=(k-{k}_{p1})(k+{k}_{p1})M({p}_{21}+{p}_{11}\alpha ) & {m}_{65}=(k-{k}_{p2})(k+{k}_{p2})M({p}_{22}+{p}_{12}\alpha ) & {m}_{66}=0\end{array}$$

## Appendix B: Stiffness Matrix of A Two-layer System

 where *G*_1_ and *G*_2_ are matrix for the first and second layer, respectively.

## Appendix C: Phase Velocity

The algorithm performs a sweep in a broad range of wavenumbers for a given frequency. A rough interval where roots exist needs to be found first and then the classic Brent’s method can be applied to accurately locates the roots. The following notations are used in the algorithm: *ϵ* for the wavenumber sweep increment; *n* for the number of iterations; *k*_0_ for the initial wavenumber, *k* for the wavenumber at the current step; ${k}^{{\prime} }$ for the wavenumber at the previous step; *f*(*k*) gives the determinant value of the stiffness matrix at wavenumber *k*; *δ* for the tolerance used to check if the determinant of the stiffness matrix is close to zero; $Brent({k}^{{\prime} },k)$ is the Brent’s method that takes an internal $({k}^{{\prime} },k)$ as input where *f*(*k*) and $f({k}^{{\prime} })$ must have different sign; *r* is the root calculated from Brent function.

The algorithm is shown as follows:

29 $$\left\{\begin{array}{l}Given\ \varepsilon ,{k}_{0},\delta ,n\\ \begin{array}{ll}for & i=1,2,\ldots n\\  & {k}^{{\prime} }=k\\  & k=k+\varepsilon \\  & {v}^{{\prime} }=f({k}^{{\prime} })\\  & v=f(k)\\  & if\ {v}^{{\prime} }\cdot v\le 0\end{array}\\ \begin{array}{llllllll} &  &  &  &  &  &  & r=Brent({k}^{{\prime} },k)\\  &  &  &  &  &  &  & if\ | f(r)|  < \delta \end{array}\\ \begin{array}{llllllllll} &  &  &  &  &  &  &  &  & \ \ return\ r\end{array}\\ end\ for\end{array}\right.$$
